# Ketal Sugar Conversion Into Green Hydrocarbons by Faujasite Zeolite in a Typical Catalytic Cracking Process

**DOI:** 10.3389/fchem.2019.00720

**Published:** 2019-11-01

**Authors:** Joana Pinto, Igor Pedrosa, Camila Linhares, Rosane A. S. San Gil, Yiu Lau Lam, Marcelo Maciel Pereira

**Affiliations:** Universidade Federal do Rio de Janeiro, Instituto de Química, Rio de Janeiro, Brazil

**Keywords:** sugar ketals, biocrude, USY, hydrocarbons, aromatics

## Abstract

Fluidized catalytic cracking (FCC) converts hydrocarbons in the presence of a catalyst based on faujasite zeolite (USY and REY). While hydrocarbon is poorly reactive, biomass and its derived compounds are highly functionalized and not suitable to a typical FCC process. To overcome this limitation biomass was first converted into a dense and stable bio-crude composed mainly of ketal-sugar derivatives by using acetone in diluted acid. Here, a representative compound of this bio-crude, 1,2:3,5-di-O-isopropylidene-α-D-xylofuranose (DX) in n-hexane, was converted by USY and a commercial FCC catalyst containing USY, at 500°C, in a fixed bed and fluidized bed reactors, respectively. Faujasite Y is very efficient in converting DX. More than 95% conversion was observed in all tests. Over 60 wt.% was liquid products, followed by gas products and only around 10% or less in coke. The higher the catalyst activity the greater the aromatics in the liquid products and yet higher coke yields were observed. In particular, simulating more practical application conditions: using deactivated catalyst in a fluidized bed reactor, improved green hydrocarbons production (mono-aromatic up to 10 carbons and light hydrocarbon up to eight carbons) and unprecedented lower coke yield (≈5 wt.%) for bio-feeds. The present results further suggest that catalyst will play a primary role to convert the bio-crude into target hydrocarbons and overcome the transition of a non-renewable to a renewable refinery feed.

## Introduction

Energy needs have been increasing substantially since the industrial revolution due to population growth and goods demand. Today oil, natural gas, and coal are the main sources for chemicals, fuel and energy production (Smil, 2004; Conti et al., 2016). But the unrestrained exploitation of these non-renewable resources almost doubled the greenhouse gas concentration in the atmosphere in the last 100 years (Russ and Criqui, 2007; North and Styring, 2015). Among several alternatives to reduce the carbon footprint, the production of green-fuels for typical refinery process can shorten our transition of a non- to a renewable refinery. As a huge ready-to-use structure for fuel products and distribution is available, and also requires simple modifications to the fuel-legislation and no adaptation in motors. Besides, simultaneously this avoids building an entire new structure (Goldthau, 2017). Thus, the conversion of biomass into regular fuel using a typical refinery is one of the most important issues that chemistry now faces (Ragauskas et al., 2006).

A major process of a typical refinery is fluidized catalytic cracking (FCC). This process produces gasoline, liquefied petroleum gas (LPG), light olefins and light cycle oil. The FCC process consists of three distinct steps: reaction, separation, and regeneration. The reaction occurs in the riser reactor in a few seconds under a reducing atmosphere due to the presence of hydrocarbons and hydrogen. After this step, catalyst and reaction products are separated by means of cyclones and a stripper. Finally, the spent catalyst with coke content in the range of 0.6 to 2.0 wt.% is directed to the regenerator vessel. The catalyst regeneration occurs at high temperature (above 700°C) and in the presence of O_2_, H_2_O, and other gases (O'Connor and Pouwels, 1994; Wen et al., 2002). The presence of steam can remarkably affect the catalyst properties (Escobar et al., 2005; Cerqueira et al., 2008) and a central point to develop laboratory test more close to typical FCC condition is to use deactivated or equilibrium catalysts. Also, the reaction-regeneration feature of the FCC makes this process eligible to co-process oil and renewable feeds (O'Connor, 2007).

The arrival of first generation of biofuels, such as bio-ethanol and bio-diesel allowed a partial replacement of fossil resources, also mitigate CO_2_ emissions. These new generation transport fuels are now facing key environmental and strategic questions, such as the fact that, they are derived from agricultural commodities that compete with food crops. In contrast, lignocellulosic biomass of forest and industrial waste are an attractive raw material to produce of renewable fuels (Ladanai and Vinterbäck, 2009; Langholtz et al., 2016). Biomass comprising cellulose, hemicellulose and lignin are commonly referred to as second generation biomass of and typically derived from non-edible residues such as bagasse and others from food industries. However, biomasses have low density and a broad diversity in functionalized components compared to hydrocarbons. Thus, it is mandatory to improve density and adjust the reactivity of any bio-feed prior to its conversion in a typical refinery process.

There are some strategies to introduce second-generation biomass in refineries (Huber et al., 2006; Serrano-Ruiz and Dumesic, 2011; Jong and Ommen, 2015). Pyrolysis and fast pyrolysis (Lappas et al., 2002; Oasmaa et al., 2003; Oasmaa and Meier, 2005; Mendes et al., 2016) have generally been accepted as primary processes for this transformation for further conversion into refinery (Adjaye and Bakhshi, 1995; Samolada et al., 1998). Pyrolysis can be performed in the presence of a catalyst (CPO) (French and Czernik, 2010; Graça et al., 2011), or post-treated by a hydro-deoxygenation (HDO) process (Saidi et al., 2014; Talmadge et al., 2014). The oils produced by these processes are typically dark, dense, viscous and composed of a complex mixture of different oxygen-containing molecules in function of the pyrolysis process, like phenols, sugars, carboxylic acids and 15–30 wt.% of water (Sfetsas et al., 2011; Silva et al., 2016). Thus, bio-oil is non-miscible with hydrocarbon, highly acid and corrosive. These bio-oils result in increase coke during co-processing with conventional feeds in FCC (Pereira and Benoit, 2013; Talmadge et al., 2014) and hence could be co-processed in very low concentration (Thegarid et al., 2014; Pinho et al., 2015). For instance, even after a severe hydrotreatment (H/C in bio-oil ≈1), high amounts of coke and gas was recorded (Table 1). The pyrolysis process solves the density, but not the reactivity problem of a bio-feed for further co-conversion at typical refinery conditions. However, bio-oil can be transformed into products by a coupling process, like hydrogen and synthesis gas (Trane et al., 2012) or fuels (Bulushev and Ross, 2011).

**Table 1:** Yield of oil products, gas, and coke used to convert bio-oil and oxygenated model compounds.

**Bio feed, catalysts, temp**.	**Liquid wt.%**	Carbon^*^ wt.%	**Gas wt.%**	**References**
Raw bio-oil, ZSM-5, 490^o^C	7	19	44	Vitolo et al., 1999, 2001
20% hydro-treated bio-oil/80% gasoil, HY 500^o^C	40	6–12	20–40	Samolada et al., 1998
Raw bio-oil	26	32	–	Vispute et al., 2010
Hydro-treated Wood Bio-oil^**^	80^***^	13	22	Vispute et al., 2010

* *Include coke, char, and Tar*.

** *100 bar of H_2_, 330^o^C, one hydrogen per carbon in feed*.

*** *Several classes of compounds*.

Recently, we proposed an alternative approach to fuel production from second generation biomass in two steps (Batalha et al., 2014). The biomass was firstly converted in mild conditions into a bio-crude (density of 1.1 gmL^−1^ and CHO composition ≈ 58, 7, and 35% by weight, respectively) combining acid catalyzed hydrolysis with organic reactions such as ketalization (Garrett et al., 2015; de Souza et al., 2017) and acetylation (Durange et al., 2015). The biocrude is composed by a mixture of isopropylidene ketals containing mono and polyshacharides-ketals (Garrett et al., 2015). For instance 1,2:3,5-di-O-isopropylidene-α-D-xylofuranose (DX) and 1,2: 5,6- Isopropylidene- α-D -glucofuranose (DG) were produced in high concentration, 50 wt.%. A later publication also applied similar condition to depolymerization wood biomass and avoided undesirable reaction of sugars (Questell-Santiago et al., 2018).

**Figure 1:**
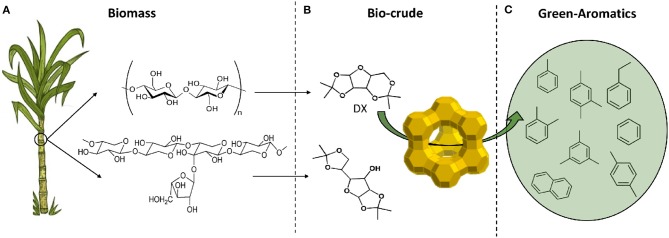
Circular economy based on second generation biomass converted into green-aromatics and -light hydrocarbons in three steps: **(A)** biomass production, sugarcane bagasse: the residue of ethanol and sugar industry, as feed; **(B)** biomass conversion into biocrude: sugar-ketal, ex. DX, DG, and polysaccharides-ketal (Garrett et al., 2015); **(C)** biocrude conversion into hydrocarbons.

Secondly, the feasibility of producing hydrocarbons, especially aromatics, using typical model components of the ketal-bio-crude such as DX and DG were shown by a simplified catalytic protocol (Batalha et al., 2014, 2016). Our approach of circular economy for producing green-aromatics can be illustrated in Figure 1.

The present work is focused on the catalyst and the catalytic testing conditions, using Y zeolite, the workhorse, active component of the FCC catalyst. First, with a fixed bed reactor, using pure USY zeolites comprehensively characterized, we showed that the DX in hexane was efficiently converted and the transformation was sensitive to catalysts properties. For example, USY was covered by silica to demonstrate that the catalyst controls the DX conversion. Then, approaching the conditions in commercial practice, a mixture of 30 wt.% of DX in n-hexane were converted in the presence of a commercial FCC catalyst, fresh or deactivated, in a fluidized bed reactor. For each process an overall view of the material balance and product distribution in gas, liquid and coke fractions, are discussed. The products distribution and catalytic properties provided insights of DX conversion into hydrocarbons.

## Experimental Section

### Catalysts

The faujazite zeolite USY (provided by Petrobras) was thermal treated in a furnace and the temperature increased from room temperature to 500 at a rate of 10°C min^−1^. At the final temperature, deionized water was pumped at 1 mL min^−1^ for 1 h. The catalyst obtained through this procedure was named USY-D500. The commercial catalyst was provided by Fabrica Carioca de Catalisadores S.A. and it was used as provided and after thermal-treatment (at a final temperature of 720°C and following the same procedure of the USY used in the fixed bed test). An additional treatment process was carried out at the USY-D500: 4 g of catalyst was dispersed under magnetic stirring in 100 mL of n-hexane and heated to 70°C under reflux. When the mixture reached 70°C, 0.6 mL of tetraethylorthosilicate (TEOS) was added. After 1 h, the mixture was filtered and the sample dried at 100°C. After 24 h, the catalyst was calcinated at 500°C for 3 h. This procedure resulted in the catalyst namely USY-D500-Silica. Prior to use, the zeolite was submitted to ion exchange using a NH_4_NO_3_ 2M solution, at 80°C, for 1 h. The ion exchange procedure was performed 4 times, followed by calcination at 450°C for 4 h under airflow. This procedure resulted in no sodium being detected in the final sample as previously reported in the literature.

### Catalytic Cracking

The n-hexane cracking in differential reactor was carried out in a high throughput unit (Ferreira et al., 2014). All experiments were conducted at room pressure (open reactor) in a continuous down flow fixed-bed micro reactor. Prior to catalyst evaluation, all the catalyst were heated simultaneously under N_2_ flow from room temperature to the desired temperature (773 K), at a rate of 10 K/min. Then the reactor flow was modified to a mixture of n-Hexane 10% v/v in N_2_ (30 mL/min) for all catalytic tests. A typical run was carried out with 0.01 g of catalyst and reaction products were analyzed on-line after three distinct times on stream (3 min, 17 min and 32 min) by gas chromatography using a Shimadzu GC-2010 (Maia et al., 2010). The activities and selectivity were presented as the average result obtained after 17 min and 32 min on stream. The error in conversion and selectivity of n-hexane test was determined in previous working and are <5 and 2%, respectively (Ferreira et al., 2014).

Reactions performed in a fixed bed reactor (Figure S1) occurred at 500°C, under atmospheric pressure and nitrogen flow (100 mL min^−1^) and 400 mg of catalyst. Prior to reaction, the catalyst was heated under a nitrogen flow (100 mL min^−1^) from room temperature to 500°C at a rate of 10°C min^−1^, remaining at this final temperature for 30 min. During the reaction, a 0.2 mL min^−1^ liquid flow of DX in n-hexane, at room temperature, was directly to the reactor entrance and mixed with N_2_ (100 mL min^−1^). For comparison, pristine n-hexane cracking was carried out with the same reaction protocol and catalyst mass. The fluid catalytic cracking unit used to convert the new bio-feed in this study is presented in Figures S2, S3 (Pinto et al., 2017). The tests were carried out by using 10 mL of feed (pristine n-hexane and mixtures of DX of 30 wt.% in n-hexane) injected over 1 min, and 20 g of the fresh and deactivated catalyst were used in the catalytic bed. Before the reaction, the catalyst was activated in a nitrogen atmosphere for 12 h at 773 K. Nitrogen flow was calibrated at room temperature, and nitrogen flow at 200 mL min^−1^ (estimated at 500°C using the state equation of ideal gas) was used to fluidize the catalyst. The reactor was operated in between 450 and 500°C considering the height of the fluidized catalytic bed (Figure S3). OPAL is a fresh commercial FCC catalyst supplied for this work by Fábrica Carioca de Catalisadores S.A., and was used with particle diameter 115–200 mesh equivalent to a particle size diameter of 0.125–0.08 mm, respectively, but the composition is unknown.

The reaction products were distributed in: liquid, gas and coke. Three tests were carried out in the presence of the catalyst USY-D500 to provided an estimative of the error (Table S1). These values were 5, 4, and 6% for gas, liquid and coke, respectively. An error of 6% for all fractions was assumed as presented in **Figure 5**. The liquid fraction was obtained through condensation (−15°C in fixed bed and −18°C in the FCC test) by means of a condenser placed right after the reactor exit. The liquid amount was obtained directly by weight difference of the condenser before and after the reaction.

The liquid faction from the cracking reactions were analyzed off-line by both GCMS and CGFID. The determination of liquid products was obtained based on GCMS and the quantification was carried out in a GCFID. The GCMS system is an Agilent Technologies 7890A CG coupled to a 5975C MS in electron impact mode, an Agilent HP-5MS column was used and the oven temperature kept at 303 K for 7 min followed by a ramp to 443 K for 40 min, helium was used as carrier gas. At the inlet, a split ratio of 20:1, 14 psi pressure and 563 K was used. The GCFID system is an Agilent Technologies 7890A GC and the same method as GCMS was used.

The liquid products were obtained by subtracting the liquid mass by the mass of non-reacted *n*-hexane (determined according Text S1). The wt.% of each aromatic compounds in the liquid fraction was obtained multiplied the FID area (%), AR_i_, by the respective chromatographic factor (*f*_*i*_) that is 0.82 for aromatic compounds. The wt.% of the remaining products were equal to each FID area (%) by assuming f_i_ = 1. Remaining products, *P*_*j*_, are determined by the sum of all olefins (containing five or more carbons), all hydrocarbons (containing five or more carbons), all light compounds (containing four or fewer carbons), heavy (assuming *f* = 1) and non-determined compounds (assuming *f* = 1). The total area of liquid products was determined by the sum of aromatics (corrected by each aromatic factor) and P_j_ (total of the remaining products), equation 1-a. The aromatic/total products ratio (S_AR_) was as presented in equation 1-b. These ratios are S_ole_ (obtained by equation 1-c), S_sat_ (equation 1-d), and S_light_ (equation 1-e). Yields were obtained by multiplying the above ratios by the mass liquid products (obtained by discounting the amount of n-hexane, not reacted).

**Table:** 

**Equation**	**Parameter in the liquid product**	**Formula**
1-a	Total liquid product, in wt.%	∑*AR*_*i*_.*f*_*i*_+*P*_*j*_
1-b	Aromatic fraction S_AR_	$\frac{\sum AR_{i}.f_{i}}{\sum AR_{i}.f_{i}+P_{j}}$
1-c	Olefin fraction S_ole_ Having more than six carbons	$\frac{\sum Olefin_{j}}{\sum AR_{i}.f_{i}+P_{j}}$
1-d	Heavy saturated hydrocarbon fraction, S_sat_	$\frac{\sum S{aturated\;Hydrocarbons\;\left(c\geq 5\right)}_{j}}{\sum AR_{i}.f_{i}+P_{j}}$
1-e	Light (saturated and olefin) hydrocarbon fraction containing four and less carbons S_light_	$\frac{\sum Hydrocarbons\;{\left(c\leq 4\right)}_{j}}{\sum AR_{i}.f_{i}+P_{j}}$

The gas products composition (H_2_, CO, CO_2_ methane, and hydrocarbon up to C_4_) were analyzed on-line using an Agilent Technologies Micro-GC 490. The amount of gas produced during the reaction was determined by the difference of water displaced during the reaction and the one in pure nitrogen flow (always quantified before reaction). The gas composition obtained was estimated using the average of 5 injections made on-line during time on stream of the reaction.

The amount of coke in the spent catalysts was determined through thermogravimetric analysis (Netzsch TG-IRIS). The samples were heated, under helium atmosphere, from 35 to 250°C at a rate of 10°C min^−1^ under N_2_ atmosphere. The temperature was kept at 250°C for 30 min, after which the atmosphere was changed to synthetic air (20.9% O_2_ in N_2_) and temperature increased to 700°C at a rate 10°C min^−1^ and afterwards it stayed on an isotherm for 30 min. The amount of coke in the catalyst corresponded to the weight lost at temperatures higher than 250°C and the coke yield was estimated comparing this value (considering the amount of catalyst in each reaction) to the total feed introduced into the reactor.

The 1,2:3,5-di-O-isopropylidene-α-D-xylofuranose (DX) was synthesized through the reaction of *D*-xylose (98.5%, VETEC, 20 g) with acetone (99%, VETEC, 400 mL) in the presence of sulfuric acid (P.A., VETEC, 16 mL). The reaction was carried out at 0°C and under mechanic stirring. The sulfuric acid was slowly added to the xylose/acetone suspension to avoid any changes to the mixture temperature. Once all acid was added, the mixture was stirred for 5 h at 0°C, and then a solution of NaOH (50 wt.%) was added until pH of 5. Complete neutralization was obtained with sodium bicarbonate (VETEC). After neutralization, the solution was filtered and all acetone removed under vacuum. The remaining liquid was dissolved in ethyl acetate (99%, VETEC) and the organic phase washed with distilled water. The solvent from the organic phase was removed under vacuum and the remaining liquid was dissolved in n-hexane (VETEC 99%). The part that was insoluble in n-hexane was discarded. N-hexane was then evaporated to obtain pure DX, which was weight (yield around 25%) and dissolved once more in n-hexane to obtain the mixtures used in the reactions (10 and 30 wt.% in n-hexane). This mixture was kept at 5°C to avoid n-hexane evaporation and DX degradation. The purity of the final compound was verified after each batch synthesis, through GCMS analysis.

## Results and Discussion

### Catalyst Characterization Used in the Fixed Bed Test

**Figure 2:**
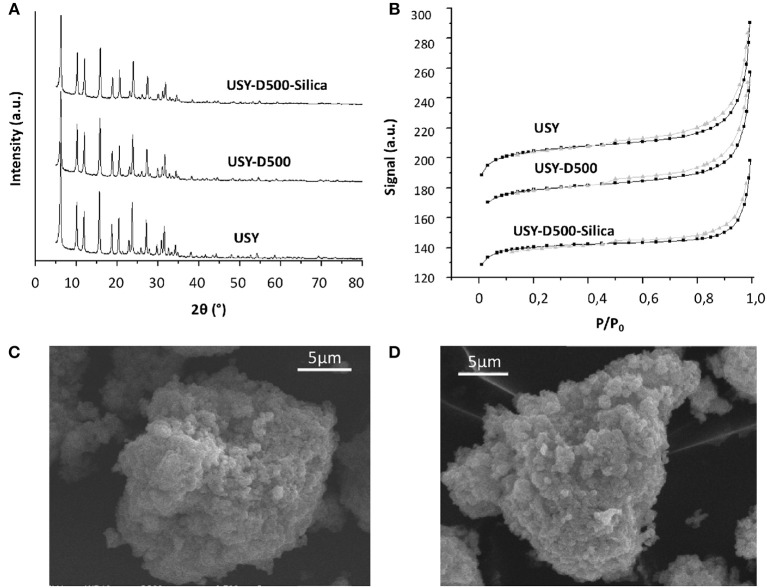
**(A)** XRD diffraction, **(B)** nitrogen isotherm for USY, USY-D500, and USY-D500-silica, **(C)** SEM of USY, and **(D)** SEM of USY-D500-Silica.

Fresh and modified USY by thermal treatment and silication are presented in Table 2. The thermal treatment in steam at 500°C slightly reduced both BET and external area compared to the USY precursor. The silication process of the USY-D500 (USY-D500-Silica) produced further decreases in the textural properties. Figure 2A shows typical type IV isotherms (IUPAC classification) of USY zeolite, with the occurrence of hysteresis cycle in the range of relative pressures 0.45<P/P_0_ <0.95 associated with capillary condensation which occurs in mesopores. The X-ray diffraction, Figure 2B, confirms the presence of Y zeolite based on Crystallography Open Database (COD).

**Table 2:** Properties of USY, USY-D500, and USY-D500-Silica.

**Catalyst**	**A_**BET**_ (m^**2**^g^**−1**^)**	**A_**ext.**_ (m^**2**^g^**−1**^)**	**Micro. vol. (cm^**3**^/g)**	XRD int. 2Θ = 23.8^°^^**^	**Si/Al_**NMR**_**	EFAL%^*^	Silica%^*^	**Si/Al _**XPS**_**
USY	627	67	0.26	100	8.7	15	4.3	1.2
USY-D500	597	64	0.25	85	14.5	40	7.6	1.0
USY-D500-Silica	522	53	0.21	77	12.7	41	11.8	1.1

* *Al as EFAL species and Si as amorphous silica are presented in molar % of aluminum and silica respectively, from MAS-NMR*.

** *Normalized using USY as 100%*.

The XRD intensity was estimated based on the peak at 2Θ = 23.8°, this signal decreased to both USY-D500 and USY-D500-Silica. Additionally, both USY and USY-500-silica showed very similar SEM microscopy (Figures 2C,D, respectively), thus the silication did not aggregate the zeolite crystals.

**Figure 3:**
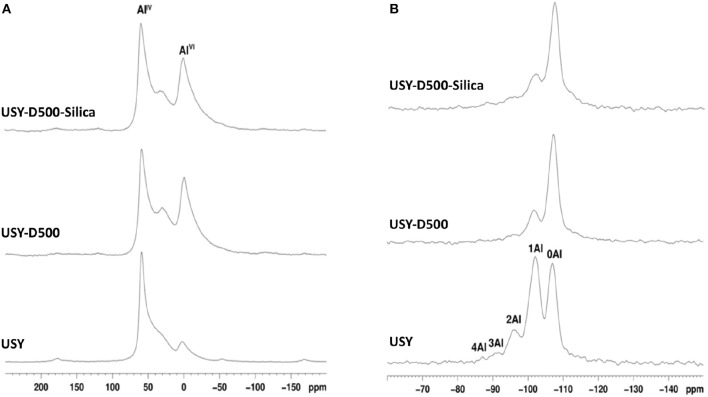
MAS-NMR spectra obtained for the catalysts studied. **(A)** ^27^Al MAS-NMR; **(B)** ^29^Si MAS-NMR (the areas of ^27^Al MAS-NMR signals are listed in Table S2, whereas the areas of ^29^Si MAS-NMR signals are listed in Table S3).

The ^27^Al RMN-MAS spectra for all catalysts, Figure 3, shows 3 signals with maxima at approximately 60, 30, and 0 ppm for all USY catalysts. These values are related to framework Al^IV^, extra-latent Al^VI^ sites with distorted symmetry and extra framework Al, EFAL (Moreno and Poncelet, 1997; Lisboa et al., 2008; Agostini et al., 2010), respectively. USY-500-silica and USY-500 catalysts showed similar intensity of both extra-latent and EFAL and higher than USY catalyst. The ^29^Si MAS-NMR spectrum, Figure 3, showed signals -110, -104, -97, and -92 ppm and the number of aluminum coordinated with silica is indicated in Figure 3B. The former is assigned to an amorphous silica phase this signal increased after thermal treatment (USY-D500) compared to USY. Thermal treatment decreased the framework aluminum, thus increasing the SAR and also increased the amount of amorphous silica on USY-D500 compared with USY. The USY-D500-Silica catalyst showed an increase in the amorphous silica but similar SAR compared to USY-D500.

The Si/Al molar ratio estimated by XPS was also presented in Table 2. This value decreased from USY to USY-D500, thus suggest that the amount of aluminum increased in the outer surface of the catalyst, most probably as extra framework aluminum species (EFAL) formed during the hydrothermal treatment. By adding silica (USY-D500-silica), the Si/Al ratio increased compared to USY-D500 confirming that siliceous species are located in the outer surface of the catalyst. The quantification of total acidity was estimated based on the total ammonium desorption as presented in Figure S4, USY, USYD500, and USY-D500-silica showed 1399, 799, and 669 μmol of NH_3_ ${\text{g}}_{\text{cat}}^{-1}$. Hence, these results confirm the intended modifications that the USY was submitted.

The n-hexane cracking was carried out in differential condition (low conversion, n-hexane/ catalyst ratio 1.18 and in the presence of 10 mg of catalyst), as presented in Table 3. The differential condition is sensitive to catalysts acidity, and a remarkable reduction (higher than 2-fold) was observed after thermal treatment of the catalysts. Further reduction was observed in the presence of USY-D500-silica compared to USY-D500. The TOF (Frequency of turnover), Table 3, was calculated based on the quantity of the framework aluminum calculated by NMR, that is, corresponding to the Brønsted sites. Roughly USY, USY-D500 showed similar TOF values (around 1), but TOF decreased 75% in the USY 500-D00Silica compared to USY. This reduction is much higher when compared to the decreased in BET area and should be related to lower accessibility of n-hexane caused by the external porous blocking by siliceous species.

**Table 3:** n-hexane cracking in differential conditions in the presence of USY catalysts used in the fixed bed tests.

**Catalyst**	**Rate_**n-C6**_ (mmol/g_**cat**_ min)**	**Conv_**n-C6**_ (%)**	**Propylene/ propane**	**TOF min^**−1**^**	**Total olefin/paraffin**
USY	2.00	9.8	1.1	1.15	1.11
USY-D500	0.81	3.9	1.6	0.74	1.37
USY-D500Silica	0.46	2.5	2.3	0.29	1.45

n-Hexane cracking in differential conditions produces exclusively light hydrocarbons (Guisnet and Pinard, 2018) as presented in a simplified reaction pathway in Figure 4. After the protolytic reaction (Kotrel et al., 2000) (in low conversion and differential conditions) n-hexane is converted into molecular propylene/propane, ethylene/butanes and ethane/butenes ratios close to one. Higher rate of hydrogen transfer reaction decreases the olefin concentration (Miyaji et al., 2015) and both propylene/propane and total light olefins/total light paraffins (in wt.%) ratio may be used to indicate the hydrogen transfer (HT) reaction. Both ratios increased from USY to USY-D500 and further increased were observed to USY-D500-Silica. As the USY catalyst is gave much higher conversion; we would only compare USY-D500 with USY-D500-Silica to avoid the conversion effect on the extent of secondary reaction of hydrogen transfer. But in this case, we observed that the silicate USY had a large increase in olefin/paraffin ratio, more than expected.

**Figure 4:**
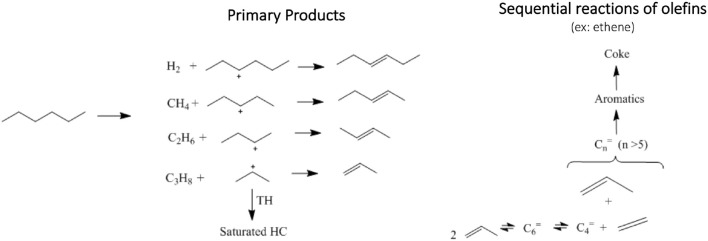
n-Hexane conversion into hydrocarbons, primary products are hydrogen, methane, light hydrocarbons up to 6 carbons (paraffins and olefins), secondary products involves a combination of several reactions, for instance olefins condensation produces larger olefins which can be sequentially converted to aromatics and coke.

### Co-conversion of 10% DX in n-Hexane in Fixed Bed Reactor

The material balances of the fixed bed tests, given by the sum of the gas, liquid, and coke fractions were higher than 94% (as presented in Table S1). The product yields distribution were hence normalized (each product was divided by the sum of all weights) as presented in Table 4.

**Table 4:** Gas, liquid, and coke yield for fixed bed catalytic test using pure n-hexane and DX 10 wt.%.

**Catalyst**	**Feed**	**Gas**	**Liquid**	**Coke**	**Conv nC_**6**_ (%)**	**Conv. DX (%)**
		**Normalized wt%**				
USY	n-C_6_	35.5	62.5	2.1	41	−
	10%DX	29.5	66.5	4.1	30	100
USY-D500	n-C_6_	27.9	70.9	1.2	29	−
	10%DX	19.9	76.9	3.2	20	100
USY-D500Silica	n-C_6_	25.2	74.2	0.7	30	−
	10%DX	13.5	83.5	3.0	11	98

Pure n-hexane showed the highest conversion in the presence of the USY catalyst, conversion decreased for the remaining catalysts. The decrease is consistent with the catalysts features as previously discussed. In the presence of 10 wt.% DX, the n-hexane conversion decreased similarly in both USY and USY-D500, by ~10%. Yet higher decrease was observed for USY-D500-Silica catalyst ~20%. In contrast, DX is almost fully converted in all tests, so its conversion not being sensitive to the modification in the USY properties. n-Hexane demands highly active catalytic sites (Haag et al., 1984; Narbeser et al., 1995), and the presence of DX could decrease the amount of available sites for n-hexane, thus suggest that the competition between n-hexane and DX put n-hexane at disadvantage.

The gas yield of pure n-hexane followed the behavior of n-hexane conversion, i.e., gas yield was higher in the presence of catalyst USY and decreased to similar values for the remaining catalysts. For the cracking of 10% DX in n-hexane, the yield of gaseous products slightly decreased compared to the cracking of pure n-hexane. In the presence of all catalysts the decrease in gas yield correlated with the decrease in the n-hexane conversion. The amount of CO and CO_2_, produced from DX are up to approximately 20 wt.%.

Coke in spent catalyst is produced by several consecutive reactions (Cerqueira et al., 2000, 2005; Bayraktar and Kugler, 2002; Occelli, 2002; Reyniers et al., 2002), coke amount in wt.% of total products reduced as the activity of the catalyst was reduced, such as by thermal treatment. USY-D500-silica catalyst produced less coke. Coke increased roughly 2 wt.% by converting 10 wt.% DX in n-hexane in the presence of all catalyst, thus deactivation did not affect the conversion of DX into coke in fixed bed reactor.

Yet, it is interesting to point out that in the MAS-NMR and XPS results indicate that the USY-D500Silica contained siliceous species that may be covering some EFAL. With less accessible Lewis sites, coke formation may be reduced compared to USY-D500 catalyst as Lewis sites can improve coke formation (Humphries et al., 1993).

The n-hexane and DX experiments were carried out in the presence of 0.5 g of catalyst and a n-hexane/catalyst ratio close to 3.6, as we aim to the verify the performance of the catalyst in full DX conversion.

Hydrogen and methane are produced in small amounts when converting pure n-hexane. With the addition of DX, n-hexane conversion decreased, hydrogen production also decreased and in contrast methane increased. Hydrogen and methane are produced from protolysis of σ C-H and C-C and bonds, respectively as presented in Figure 4, thus producing a carbenium ion that originates a catalytic cycle (Louis et al., 2010). It is important to point out that DX is more active than n-hexane, as in all tests it was fully converted. As methane did not follow the decreased in the n-hexane conversion, DX could contribute to methane, like for example by the protolytic reaction of isopropylidene groups.

The DX deoxygenates produce CO and CO_2_, also carbon dioxide are almost double of carbon monoxide in all tests. A higher concentration of CO_2_ compared to CO indicates that less carbon (from DX) is loosed during the decarbonization and the decarboxylation process. These products increased from USY to less active catalyst. This behavior is related to lower n-hexane conversion, thus the relative concentration of CO and CO_2_ increased.

The main products of n-hexane are light olefins and saturated hydrocarbons. The former are more reactive than n-hexane and can undergo sequential reaction like hydrogen transfer reaction, alkylation, cyclization, producing a broad type of products. For instance, aromatics were produced in the presence of high amount of catalyst and absent in the n-hexane cracking in differential conversion. Thus, the propylene/propane ratio decreased 3-fold in these experiments compared to the ones carried out in differential conditions. On DX and n-hexane test condition the propylene/propane ratio for pure n-hexane was 0.3, as propylene is consumed in sequential reaction, this ratio slightly increased to 0.47 in the presence of USY-D500. Less active catalyst decreases hydrogen transfer reaction, as fewer acid sites are available for sequential reactions. The propylene/propane and olefin/total saturated hydrocarbons ratios increased for DX and n-hexane mixture compared to pure n-hexane to both USY and USY-D500 (Table 5). Further increase is observed in the presence of USY-D500-Silica compared to USY-D500.

**Table 5:** Selectivity (Wt.%) of the gaseous products for USY, USY 500, and USY 500-Silica^*^.

		**H_**2**_**	**CH_**4**_**	**CO**	**CO_**2**_**	**${\text{C}}_{3}^{=}$/C_**3**_**	**Olef/sat**
USY	Hexane	0.05	0.9	–	–	0.34	0.31
	10% DX	0.02	1.1	1	2.2	0.43	0.37
USY 500	Hexane	0.04	0.6	–	–	0.47	0.40
	10%DX	0.02	1.3	1.6	3.6	0.63	0.50
USY 500Silica	Hexane	0.03	0.8	−	−	0.55	0.48
	10%DX	0.02	1.7	2.3	4	0.69	0.55

* *Detail gas products in Table S4*.

The increased in light olefins selectivity in the presence of the less active catalyst and particularly in the presence of DX is discussed considering that these products are related exclusively to n-hexane then included the contribution of DX. The olefin/paraffin ratio and n-hexane conversion showed opposite tendencies and as the conversion of n-hexane also decreases in the presence of DX prevents a more conclusive analysis. However, the effect in olefins formation taking into consideration that DX is present in small amounts could be related to some contribution of DX to these products or a mutual interaction between DX and n-hexane. Related to the latter proposition the olefin/paraffin ratio could be affected by decreasing olefins consumption (for instance by a competition with acid sites with oxygenated compounds), or by increasing olefin production due to hydrogen transfer reaction (between the hydrocarbon and the oxygenated derivatives).

**Figure 5:**
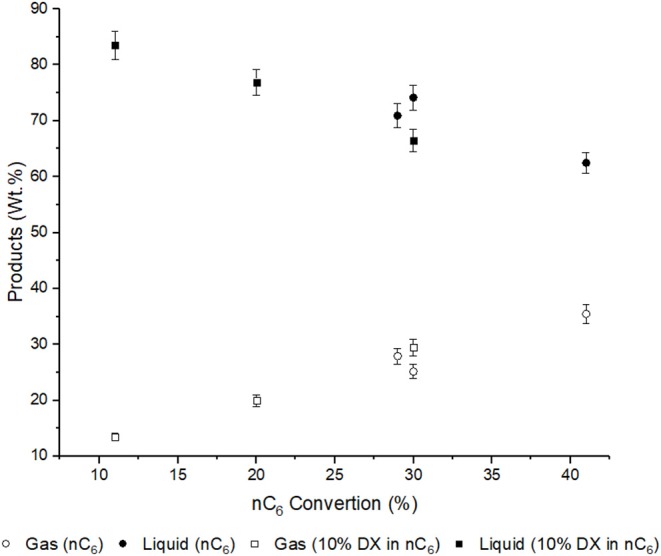
Liquid and gas yields and n-hexane conversion. Error bars (6% of the gas and liquid yield value) are introduced (Table S1).

The liquid yield is affect by mainly two features, non-reacted n-hexane and amount of aromatics. The liquid yield decreased with n-hexane conversion. It was lowest for USY, as this was a more active catalyst, and was highest in conversion of DX in n-hexane over USY-D500-Silica, Figure 5.

The liquid fraction was analyzed qualitatively to verify oxygenate compounds (GCMS) and quantitatively for hydrocarbons (GCFID). As explained in the experimental section the reaction products are grouped by class and the focus here was to show that DX is mainly transformed to aromatics (Table 6). Pure n-hexane converted in the presence of USY produced mainly saturated hydrocarbons (in the liquid fraction light hydrocarbons are solubilized and paraffin and olefins up to seven carbons were observed) followed by a significant amounts of aromatics. The mixture of 10 wt.% DX converted in the presence of USY more than doubled the aromatic/total liquid products compared to pure n-hexane. This marked increase in aromatics when DX in n-hexane was used instead of pure n-hexane was observed for all other zeolites. This indicates a strong tendency in generating aromatics from DX.

**Table 6:** Aromatic selectivity, percentage of n-hexane conversion for the cracking tests with pristine n-hexane and for the mixture of 10%DX in n-hexane.

	**USY**		**USY-D500**		**USY-D500-Silica**	
S_Ar_%	29	57	22	41	32	51
AR yield %	1.4	3.8	2.9	4.6	4.0	8.2
DX conv. %	–	97	−	97	–	95
nC_6_ conv. %	41	30	29	20	30	11

When converting DX and n-hexane in the presence of USY-D500 catalyst the aromatic/total liquid products decreased around 30% compared to USY. Aromatics are secondary products of n-hexane and produced from cyclization, hydrogen transfer and light olefins re-conversion. Thus, fewer aromatics are produced in the presence of a less active catalyst. Similar explanations could be applied for the decrease in aromatics from USY to USY-D500Silica observed in conversion of DX and n-hexane mixture.

But from the XPS and MAS-NMR results: the effect of blocking some EFAL sites and reducing diffusivity due to silication could increase bimolecular reaction thus enhancing aromatic production of USY D500-Silica compared to USY-D500.

**Figure 6:**
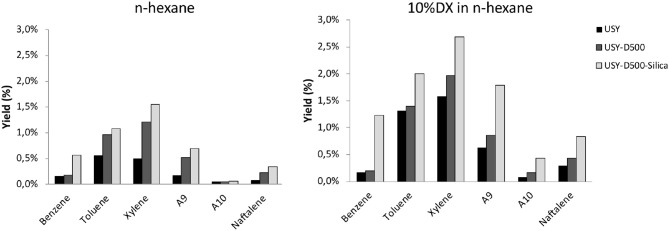
Mono-aromatic and naphthalene yield (in wt.%) observed in the presence of USY, USY-D500, and USY-D500-Silica.

The yield of each aromatic compound is presented in Figure 6. n-Hexane produced mainly, toluene and xylene regardless the catalyst. In general similar aromatic distributions are observed for USY and USY-D500 while USY-D500-Silica enhance benzene and three-methyl-benzene. Further, the aromatic distribution is quite similar to n-hexane and DX n-hexane mixture.

The modification of USY by thermal treatment combined with silication leads to significant change in the catalyst properties compared to fresh catalyst that resulted in a improvement in green aromatic yields (higher than 2-fold). However, coke formation from DX was apparently less affected by the type of catalyst, al least by converting 10 wt.% DX in n-hexane in fixed bed tests.

In short, Y zeolite converted efficiently DX, a model compound of the bio-crude, mainly into a liquid product in particular aromatics. Also, part of DX left as light gases, CO, CO_2_, and coke. The gas and coke decreased while the aromatic liquid product increased when the activity of USY was reduced. To explore these results in selectivity for applications, we have to reduce more drastically the activity of the USY and increase the catalyst to feed ratio. The later to ensure maximum removal of the oxygenated groups. Hence, we resort to using a fluidized bed reactor and real commercial FCC catalyst containing USY zeolite. Testing the adequately hydrothermally treated FCC catalyst in fluidized bed indeed bring us to a regime close to conditions in FCC in industrial practice.

### Conversion DX in n-Hexane Under Fluidized Catalytic Cracking Conditions With Deactivated Commercial FCC Catalyst

Herein we demonstrate an example of DX and n-hexane conversion in a typical condition. A mixture of 30 wt.% of DX in n-hexane was converted by a commercial FCC catalyst fresh [Cat or hydrothermally treated at 720°C (Cat D)]. The properties of the commercial FCC catalyst are presented in Table 7, and XRD for both catalysts are presented in Figure S5.

**Table 7:** Properties of the FCC commercial catalyst, Cat as provided by FCC S.A.

**Properties**	
Area BET (m^2^/g)	250
Density (g mL^−1^)	0.82
Pore volume (mL g^−1^)	0.35
Re_2_O_3_ (wt.%)	0.54
Al_2_O_3_ (wt.%)	59.2
Fe (wt.%)	0.62
Na (wt.%)	0.31
P_2_O_5_ (wt.%)	0.07

The material balance of the fluidized catalytic cracking is obtained as in the fixed bed test and was in the range of 91–106. The products yields were done by normalizing the coke, gas and liquid fractions to 100%, likewise in the fixed bed test.

Pure n-hexane showed the highest conversion with fresh Cat and decreased with deactivated catalyst (Cat D) as expected (Table 8). In mixture with 30% DX, n-hexane decreased 20% in conversion reacting on fresh catalyst and this decrease was notably more drastic on Cat D.

**Table 8:** Conversion of n-hexane and DX and global product distribution in the fluidized bed test using pure n-hexane and DX 30 wt.%.

	**Feed**	**Yields wt% (normalized)**			**Coke on cat. (wt.%)**	**Conv. nC_**6**_ (%)**	**Conv. DX (%)**
		**Gas**	**Liquid**	**Coke**			
Cat	n-C_6_	29	70	6.6	2.1	40	–
	30% DX	30	60	13.2	4.6	32	99.7
Cat D	n-C_6_	9	90	1.8	0.6	19	–
	30% DX	23	78	3.9	1.4	11	99.6

Thus, n-hexane conversion was affected by the type of the catalyst and DX. In contrast, DX was almost fully converted in all tests. The more drastic decrease in n-hexane conversion in the case of mixture with DX over deactivated catalyst suggest that DX was more successful in competing for the active sites of the catalysts than n-hexane.

The conversion of pure n-hexane with fresh Cat produced high amount of coke. Further increase in the coke yield was observed by converting the mixture of 30 wt.% of DX in n-hexane. High coke yield is a consequence of excessive reconversion of the products and a fresh catalyst did not represent a typical FCC condition. Thus, after hydrothermal treatment, Cat D obtained gave 3-fold decrease in coke yield. At any rate, considering this coke yield is from test using a laboratory reactor, it was remarkably and encouragingly low comparing to values presented in Table 1. More discussions will be put forward later.

Methane, hydrogen, CO, CO_2_, propylene/propane and olefins/paraffins ratios are presented in Table 9 and the detail gas products in Table S5. Pure n-hexane produced low hydrogen and methane in both Cat and Cat-D. Both tests showed similar hydrogen, methane and propylene/propane ratio. The methane and propylene/propane ratio doubled when converting 30 wt.% DX in n-hexane compared to pure n-hexane in the presence of Cat. In the presence of Cat D these properties increased 4-fold compared to pure n-hexane. These results support that DX contributed to methane and light olefins formation, similarly as observed in the fixed bed tests. The CO/CO_2_ ratio was close to one while in the fixed bed tests was around 0.5.

**Table 9:** Hydrogen, methane, CO, CO_2_, propylene/propane, and light olefins/light paraffins ratios (in wt.% of total gas) in the presence of Cat and Cat D.

**Catalyst**	**Feed**	**H_**2**_**	**CH_**4**_**	**CO**	**CO_**2**_**	**Propylene/ propane**	**Olefin/ paraffin**
Cat	n-hexane	0.1	3.4	0.0	0.0	0.33	0.41
	30% DX	0.1	6.4	5.0	6.0	0.58	0.68
Cat D	n-hexane	0.1	3.0	0.0	0.0	0.38	0.47
	30% DX	0.1	11.2	12.0	10.0	1.67	2.03

**Figure 7:**
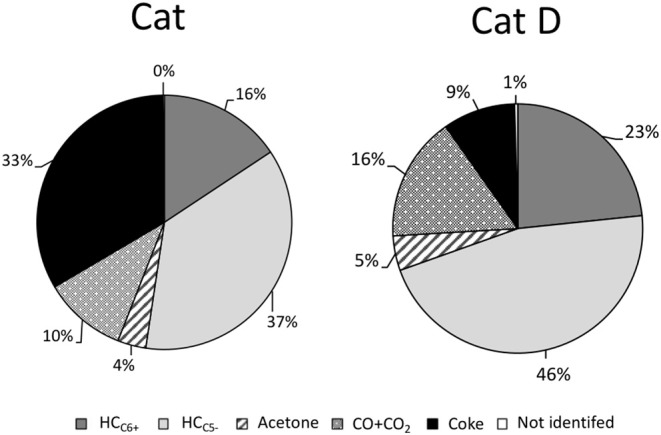
DX distribution in aromatics, light hydrocarbons in the gas phase, CO + CO2, acetone, and non-identified products.

The amount of oxygen contained in both CO and CO_2_ was estimated for Cat and Cat D as 20 and 30%, respectively. The amount of gas products in the DX and n-hexane mixture was discounted from CO and CO_2_ and also considering that n-hexane behaves equally when pure and mixture with DX. Firstly, DX contributes to light hydrocarbons in the gas phase and mainly to light olefins, judging by the increased in the olefins in the presence of DX and as sequentially presented in Figure 7.

The liquid fraction is separated into six main classes as presented in Table 10. The aromatic/total liquid products ratio S_Ar_ increased 6-fold by converting DX and n-hexane in the presence of Cat compared to pure n-hexane, and further increase was observed in the presence of Cat D. By contrast, the fraction of light saturated hydrocarbons decreased in converting DX and n-hexane in the presence of Cat compared to pure n-hexane, and further decrease was observed in the presence of Cat D. Non-aromatic was produced in low amount in all tests. Light products decreased 2-and 4-folds in the presence of DX for Cat and Cat D, respectively. Acetone was detected and quantify whenever DX was being transformed. Finally non-identified compounds were recorded in the presence of DX. In short, the outstanding observation in the liquid fraction was the high contents in aromatic products. The aromatic distribution is presented in Table 11 and in all cases the main products were xylene and toluene followed by C_3_-phenyl, benzene and naphthalene.

**Table 10:** Liquid products distribution with Cat and Cat D.

**Class/total products**	**Cat**		**Cat D**	
	**n-C_**6**_**	**30% DX in n-hexane**	**n-C_**6**_**	**30% DX in n-hexane**
S_Ar_%	7%	41%	6%	56%
S_nonAr_%	4%	6%	9%	6%
S_light_ (C5^−^) %	89%	39%	84%	22%
S_Heavy_ %	0%	3%	0%	3%
S_Acetone_ %	0%	6%	0%	6%
S _Noidentified_ %	0%	11%	0%	12%
n-hexane conv. %	40%	32%	19%	11%

**Table 11:** Aromatic distribution with Cat and Cat D.

	**Cat**		**Cat D**	
	**n-hexane**	**30% DX**	**n-hexane**	**30% DX**
Benzene	1.2	5.7	0.0	7.0
Toluene	1.2	18.0	3.4	22.9
Xylene	4.2	13.7	2.8	21.3
3Mbenzene	0.8	3.8	0.0	5.0
4Mbenzene	0.0	0.0	0.0	0.0
Naftalene	0.0	2.8	0.4	2.9

### Considerations on Potential in Co-processing This New Feed With Gasoil

#### The Coke Issue

The coke in the spent Cat D showed a value similar to a typical spent FCC catalyst converting hydrocarbon feed (in general around 1–1.5 wt.%). This value is remarkably lower compared to pyrolysis bio-crude converted in the presence of an acid catalyst in typical cracking conditions (Fogassy et al., 2011; Graça et al., 2013; Pereira and Benoit, 2013).

A commercial FCC unit operates in the temperature range of 500–535°C and the reaction occurs within short contact times, i.e., 3 to 7 s (Haag et al., 1984; O'Connor, 2007). In our tests for Cat and Cat D, the catalyst/feed ratio was three times lower and the contact time was 60 s. These condition are regularly applied in laboratory fluidized units and increasing the coke yield up to 3-folds compared to the FCC unit operating in typical condition (Occelli, 2002; Cerqueira et al., 2005). Hence, the coke yield of 1.4% is expected to show further reduction in a FCC unit- to an unprecedented low value, most probably similar to those observed for hydrocarbon feed like heavy gasoil. This scenario supports that biocrude based on ketal-sugar derivative could be co-converted in higher concentration with gasoil. In contrast, the co-process of pyrolysis oil in FCC increases coke and gas compared to hydrocarbons (Table 1). Also, lower carbon is incorporated in the final products (around 20–30%) and phenol derivatives and not fully converted (Pinho et al., 2015). Thus limits the amount of this bio-feed around 10 wt.% during co-process with gasoil feed in the FCC process (Pinho et al., 2015).

#### Bio-Crude to Hydrocarbon: Carbon-Hydrogen Efficiency, Example From DX

For a first approach, the products yields from DX were estimated assuming that n-hexane behaves equally when pure or mixed with DX. Hence, the contribution of n-hexane could be subtracted from total product based on the observed conversion of n-hexane and the product distribution when it is converted pure. The results are presented in Figure 7. They are grouped into green hydrocarbons containing six or more carbons, C_6+_ (aromatics, naphthalene, olefins and paraffins); hydrocarbons containing five or less carbons, C_5−_ (methane, paraffin, and mainly olefins); acetone, CO + CO_2_, non-identified compounds and coke.

In the presence of fresh catalyst 53% of DX was converted into green hydrocarbon (C_6+_ + C_5−_) and 1/3 of DX was converted to coke. Besides acetone, CO and CO_2_ were formed. The green hydrocarbons in carbon and hydrogen bases of DX are estimated dividing 53 by 65 (35 wt.% of DX is oxygen) and resulted in 81%. In the presence of a more representative catalyst of a typical FCC process, coke yield remarkably decreased (almost 4-folds), and around of 69 wt.% of DX was converted into green hydrocarbons (C_6+_ + C_5−_). This value is similar to the carbon and hydrogen contents in the DX (65 wt.%).

To interpret the above findings we noted, first, that higher green hydrocarbons yield were observed for both fresh and deactivated catalysts than expected since a considerable amount of carbon from DX was loss as coke, CO and CO_2_.

Second, it is important to point out that the hypothesis of DX distribution into products was done assuming that n-hexane behaves equally when converted pure or during co-process. Bimolecular reactions like alkylation, condensation and hydrogen transfer reaction are well-documented in hydrocarbon chemistry (Watson et al., 1997; Miyaji et al., 2015). For co-feeding DX and n-hexane, DX is more reactive than n-hexane and could de assumed to convert first. Then n-hexane (or its products) could undergo reactions with products from DX. For instance, the presence of DX could increase the hydrogen transfer reaction between n-hexane (or its products) and products derived from DX. This scenario could enhance the contribution of n-hexane to light olefins and aromatics during co-process.

Thus, on one hand, higher amount of carbon from DX is converted into hydrocarbons but the assumptions for the previous calculation of C-H efficiency are rough approximations. On the other hand, the possibilities that of the co-processed hydrocarbon participating in the overall conversion motivates further studies on the effect of the co-feed hydrocarbon and catalyst properties. Higher amount of bio-feed maybe able to be co-processed and better product slates may be obtained.

It is also important to point out some consideration on the reaction pathway of DX into aromatics. Olefins selectivity increase in the presence of DX. Therefore, the inter-conversion of olefins could respond partially to the aromatic formation, likewise in hydrocarbon chemistry (Liu et al., 2014). However, we cannot rule out a direct conversion of DX into aromatic without go through light olefins. A detailed work devoted to mechanism has yet to be performed in further works.

#### A Consideration on the Operation and Results of the Two Different Tests: FB and FCC

It is opportune to compare the results obtained using the simple fixed bed tests (FB) with that of the fluidized bed tests (FCC), even though it is not our intention to compare the operations of the two different units with distinct running conditions. Table 12 below first summarizes the operational parameters of the two tests, FB and FCC. We would like to point particularly the advantage of the FCC: that one could operate with a high catalyst to feed ratio. Hence, one obtains results from catalysts that are much less active per unit mass. If one uses the same catalyst to feed ratio in the present FB set up, diffusional and pressure drop problem will be encountered using powder catalysts.

**Table 12:** A brief comparison between the operational parameters and results on DX conversion for the fixed bed (FB) and fluidized bed (FCC) tests.

	**Fixed bed: FB**	**Fluidized bed: FCC**	**Observations**
**On operational parameters**			
Feed rate in DX	0.34 g/g cat min	0.36 g/g cat min	Similar
T° and pressure	500°C, atmospheric	450–500°C, atmospheric	Similar
Catalyst/ feed ratio (g/g relative to DX)	0.2 *(Diffusion and pressure drop problem if increased)*	3 (*Cat/feed ratio 5 or above routinely practiced)*	Advantage for FCC
Execution time	Short, 3 h per sample	One day per sample	Advantage for FB
Material balance	Satisfactory	Satisfactory	Similar
**On dx conversion, findings**			
Performance of Y zeolite	Near total conversion of 10% DX in n-hexane	Near total conversion of 30% DX in n-hexane	FCC confirmed FB on the efficiency of Y zeolite in converting DX
Aromatics in liquid	Indication of DX increase aromatics	Marked increase in presence of DX	FCC clear confirmation of FB results
Major aromatics	Benzene, toluene and xylenes	Benzene, toluene and xylenes	Confirmation of results
Selectivity in coke	Not clear due to catalyst variation	Decrease in less active catalyst	FCC: more information
Gas amount and composition	CH_4_ increased, CO and CO_2_ formed with DX feed	Same observation, but the CO/CO_2_ ratio varied	The difference in CO_2_/CO ratio still has to be interpreted
Sensitive to catalyst properties	Both showed sensibility to catalyst properties, such as acidity		
	Additional results of variations due to different zeolites recorded in FB (de Souza et al., 2017)		

First, from the fixed bed tests we observed that Y zeolites are effective in transforming DX into useful products, especially benzene, toluene and xylene, with only small generation of coke. More interesting is the observation that the less active the USY zeolite, more selective it is for aromatics and produces less light gases. The tendency of yielding less coke is observed, but it is not very sensitive to the properties of the catalyst for the samples used.

This prompted the use of much less active catalyst for its selectivity. But we still wanted to convert as much DX and remove as much oxygenates as possible. Using the FCC reactor one compensated the conversion of the low activity catalyst by increasing its amount (which was no longer constrained). As a consequence of both of these factors, as shown in Table 12, very high amount in aromatics and low coke yield was attained with almost complete conversion of DX. Needless to say, this test condition can be more readily related to industrial applications.

#### On Catalysts Deactivation and Optimization

The deactivated catalyst (Cat D) still fully transformed all DX in the feed, so it was reasonably active. However, a significant amount of light hydrocarbons and acetone were found. Yet, it was important to point out in this specific test that the catalyst to oil ratio was only <2.5 (since 20 g of catalyst was used for 10 ml feed, taking the density of the feed as 0.8 g/ml.) while in typical FCC operation in practice, the catalyst to oil ratio was easily above 5. That is, there is a lot of room to operate the deactivated catalyst to further convert light olefins, acetone and most probably, the non-identified products that could be oxygenated intermediates.

Moreover, the tests showed that the transformation of DX and n-hexane mixtures are responsive to the catalyst properties. Indeed, the catalyst activity could still be increased by changing the catalyst composition, such as the amount of zeolites and zeolite type. Besides, there is a competition for an acid site in the presence of a less active catalyst (in the co-cracking). Two points deserve consideration. Firstly n-hexane is a product of the FCC process and not converted in the presence of an equilibrium FCC catalyst, thus remarkable less reactive than large hydrocarbons. Thus, for converting DX in the presence of a gasoil feed composed of larger hydrocarbons it is expected less decrease in the gasoil conversion by the presence of DX. Secondly, mixtures of DX and light hydrocarbons could be used itself as a process for producing green-hydrocarbons, thus light hydrocarbons (like n-hexane) should be used as a solvent for DX in the presence of less active catalyst. Further, we would also point out a different consideration in the mode of operation for producing light olefins and/or aromatics thanks to the flexibility and adjust the reactivity of DX to be transformed in typical FCC process.

## Conclusion

The present work is a proof of concept that biomass can be converted into green hydrocarbon, in typical refinery conditions, by using the approach of protective reaction.

Using a fixed bed reactor, Y zeolite showed efficient conversion of a model compound of bio-crude, DX. The gas and coke decreased while the aromatic liquid product increased when the activity of USY was reduced. Aromatics were the main product.

These results were confirmed and extended to a regime close to conditions in FCC in industrial practice. Mixtures of DX (up to 30 wt.%) in n-hexane, were converted by fresh and deactivated commercial FCC catalysts in fluidized process. The deactivated catalyst increased the green hydrocarbons products and decreased coke yield compared to the fresh one. DX was mainly converted into green aromatic and light hydrocarbon (mainly olefins) yet gave only a very small amount of coke in the presence of a deactivated catalyst.

The test also showed that the transformations of DX and n-hexane mixtures were responsive to the catalyst properties. Thus, it is expected that catalyst will play a key role in further improving the conversion of sugar-ketal derivatives in the refinery.

## Data Availability Statement

All datasets generated for this study are included in the article/Supplementary Material.

## Author Contributions

JP made the catalytic tests in fixed bed and fluidized bed, the calculations, and the development of the article. IP carried out tests in the fixed bed. CL performed n-hexane cracking tests. RS performed and interpreted the NMR analyzes. YL assisted the interpretation of the results. MP writes the manuscript and manages the work.

### Conflict of Interest

The authors declare that the research was conducted in the absence of any commercial or financial relationships that could be construed as a potential conflict of interest.
