# Supplementary material for: Ketal Sugar Conversion Into Green Hydrocarbons by Faujasite Zeolite in a Typical Catalytic Cracking Process
Source: Front Chem. 2019 Nov 1;7:720. doi: 10.3389/fchem.2019.00720 (PMC6839337; doi:10.3389/fchem.2019.00720)
Supplement: Supplementary file 1 [file Table_1.DOCX]

**Supplementary Information of “Faujasite Zeolite, the key to control the ketal sugar conversion into green aromatics in typical catalytic cracking process”.**

Joana Pinto, Igor Pedrosa, Camila Linhares, Rosane A.S. San Gil, Yiu Lau Lam and Marcelo Maciel Pereira*

Instituto de Química – Universidade Federal do Rio de Janeiro, Rio de

Janeiro, RJ, Brazil - * [maciel@iq.ufrj.br](mailto:maciel@iq.ufrj.br)

**Supplementary Figures**

**Figure S1:** experimental hardware of the fix bed catalytic unit. Before the reaction, the catalyst is dried under an inert atmosphere of N_2_ at atmospheric pressure and under a nitrogen flow (100 mL.min-1), measured by a flow controller (1). The reactor (made of gass) with the catalyst is embedded is heated by a furnace (2), which is heated to 500 ° C at 10 ° C min ^-1^, heating during 30 minutes at final temperature is enough to ensure a complete drying of the catalyst. After the complete drying procedure the liquid mixture, composed of DX (10, 20 and 30 wt.%) in n-hexane is injected by a syringe pump (3), at a flow rate of 0.2 mL.min-1, and mixture with nitrogen in the entrance of the reactor. Following the reactor is a condenser (4), where the less light products constituting the liquid fraction are condensed at -15 ° C. Light products that were not condensed follow the line and are analyzed by a Micro-GC (5). Subsequent to the micro GC is positioned a two-way valve (6), in which the gas can be sent to vent or determined its volume by the displacement of liquid, which is discounted from the test with pure nitrogen.

**N_2_**


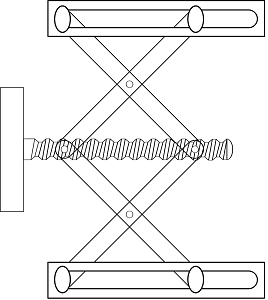

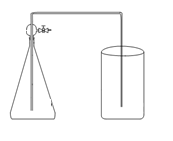


**Vent**

**Purgega**

3

2

5

6

**Micro GC**

1

4

**Volume of gas measured by water displacement**

**Flow control**

**Figure S2:** Experimental hardware of the fluidized bed catalytic unit. The feed (pure n-hexane, or mixed with 30 wt.%DX) is pumped in 1 and mixture with nitrogen, the mixture containing n. Nitrogen flow (used to fluidized the catalyst) is controlled by the flow control 3, the liquid and nitrogen are introduced in the reactor 6. The reactor is divided into two parts, the reaction section and the expansion chamber containing a baffle to prevent catalyst drag. The reactor is heated by the furnace (5) and the temperature profile is shown in Figure S3. After the reaction the products are condensed (7) at -16 ° C (9). The lighter fractions (not condensed) proceed to a glass device (12) to determined the gas volume by liquid displacement. The nitrogen gas flow is calibrated before the reaction by a volumetric calibrated pipet (10) and after connect to exhaust gas line by using a Vent (11).


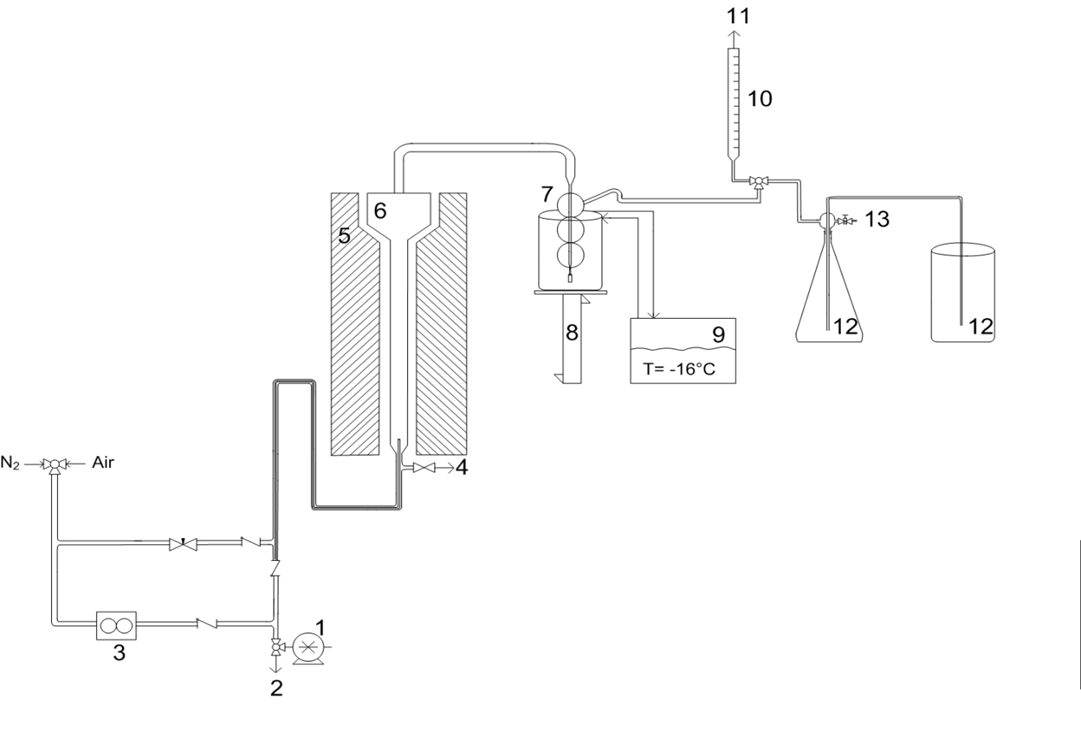


**Figure S3:** Profile of the fluidized reactor temperature.

**
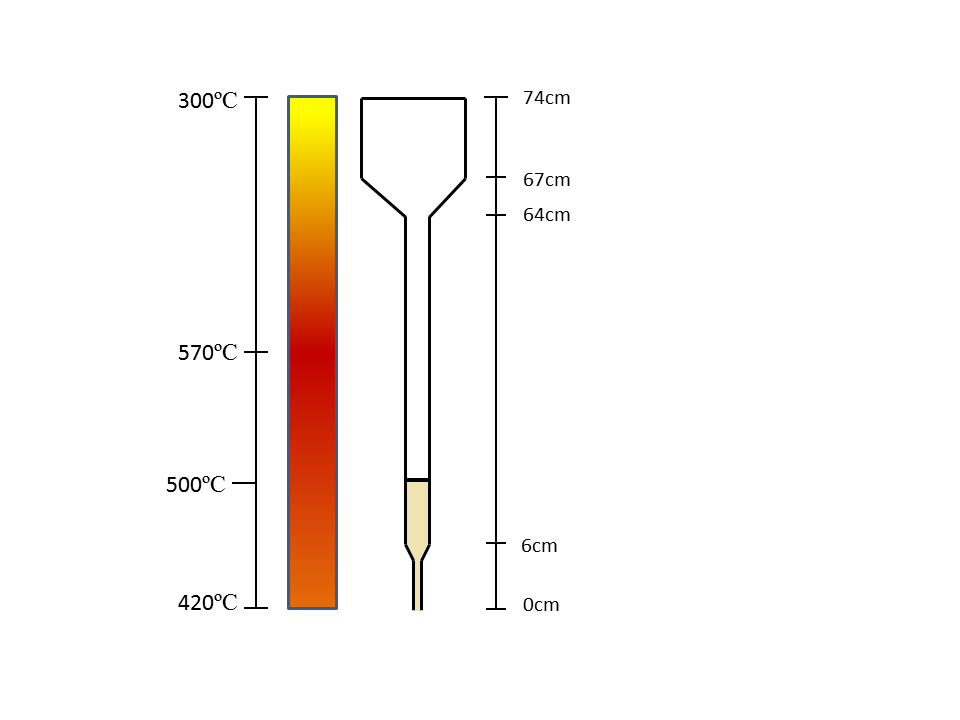
**

**Figure S4:** Profile of ammonium desorption

**
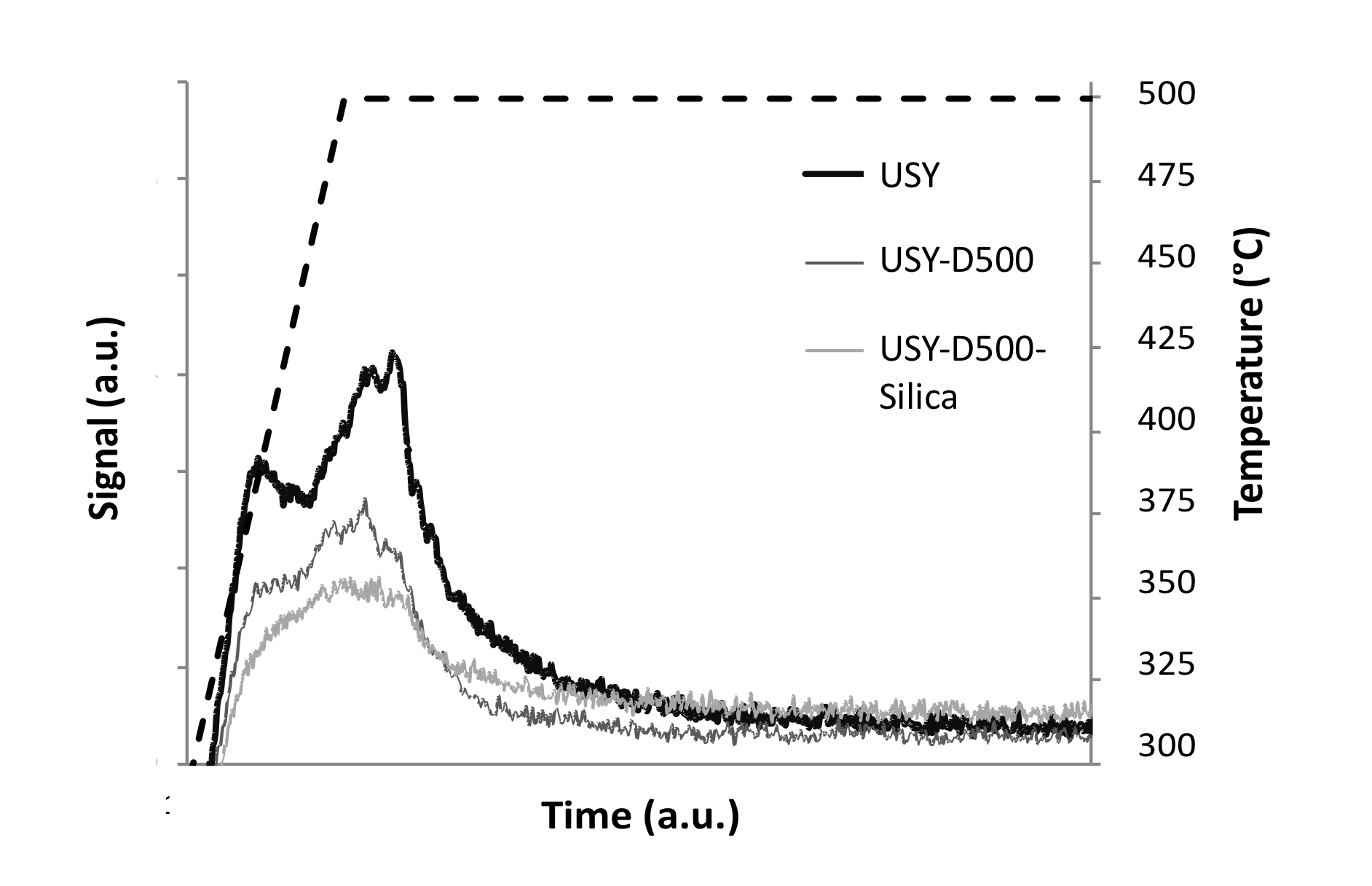
**

**Supplementary Tables**

**Table S1:** The material balances of the fix bed tests. Also, the reproducibility of the catalytic tests of DX and n-hexane. The catalyst USY-D500 was used and three tests were carried out. Error was calculated based on the average of each fraction and were lower than 6% for all fraction.

|  |  | **Gas** | **Liquid** | **Coke** | **Total** %(m/m) |
| --- | --- | --- | --- | --- | --- |
| **USY** | n-C_6_ | 34 | 61 | 2.1 | 97 |
|  | 10%DX | 28 | 65 | 4.1 | 97 |
| **USY-D500** | n-C_6_ | 25 | 68 | 1.2 | 94 |
|  | 10%DX | 17 | 74 | 3.2 | 95 |
|  | 10%DX | 16 | 78 | 3.0 | 97 |
|  | 10%DX | 18 | 73 | 3.5 | 95 |
| **Error (%) of each fraction based on USY-D500** | | **5%** | **4%** | **6%** | **1%** |
| **USY-D500-Silica** | n-C_6_ | 24 | 73 | 0.7 | 98 |
|  | 10%DX | 13 | 83 | 3.0 | 99 |

**Table S2:** chemical shifts and the areas of each peak of RMN-MAS de ^27^Al.

|  | **δ (ppm)** | |  | | **Area (%)** | |
| --- | --- | --- | --- | --- | --- | --- |
|  | Al^IV^ (structural) | Al^VI^ (non structural) | | Al^IV^ (structural) | | Al^VI^ (non structural) |
| **USY** | 59,5 | 2,9 | | 63,5 | | 15,4 |
|  | 31,2 |  |  | 21,1 | |  |
| **USY-D500** | 59,5 | 0 | | 38,7 | | 39,9 |
|  | 30,6 |  |  | 21,5 | |  |
| **USY-D500-Silica** | 58,9 | 0 | | 40,8 | | 40,9 |
|  | 37,0; 32,4; 28,3 |  |  | 18,4 | |  |

**Table S3:** Chemical shifts and the areas of each peak of RMN-MAS de ^29^Si.

|  |  | **USY** | **USY-D500** | **USY-D500-Silica** |
| --- | --- | --- | --- | --- |
| **Q^4^(4Al)** | δ (ppm) | 87,1 | -80,8 | -84 |
|  | Area(%) | 1,9 | 1,8 | 1,9 |
| **Q^4^(3Al)** | δ (ppm) | 90,7 | -92 | -92 |
|  | Area(%) | 4 | 2,3 | 3,2 |
| **Q^4^(2Al)** | δ (ppm) | -95,8 | -96,5 | -96,1 |
|  | Area(%) | 12,7 | 6,6 | 7,7 |
| **Q^4^(1Al)** | δ (ppm) | -102,1 | -101,9 | -101,9 |
|  | Area(%) | 43,5 | 23,6 | 22,8 |
| **Q^4^(0Al)** | δ (ppm) | -107 | -107,4 | -107,5 |
|  | Area(%) | 33,7 | 58,1 | 52,5 |

**Table S4:** Selectivity of the gaseous products each catalysts USY, USY-D500 and USY-D500-Silica

|  | |  | H2 | CH_4_ | C_2_H_4_ | C_2_H_6_ | C_3_H_6_ | C_3_H_6_ | C_4_H_10_ | C_4_H_8_ | CO | CO_2_ |
| --- | --- | --- | --- | --- | --- | --- | --- | --- | --- | --- | --- | --- |
| **USY** | Hexane | | 0.05 | 0.9 | 3.2 | 1.8 | 55.5 | 18.6 | 18.5 | 1.4 | - | - |
|  | 10% DX | | 0.02 | 1.1 | 3.8 | 1.3 | 44.4 | 19.1 | 22.3 | 2.4 | 1.0 | 2.2 |
| **USY-D500** | Hexane | | 0.04 | 0.6 | 3.5 | 1.6 | 46.6 | 22 | 21 | 2.3 | - | - |
|  | 10%DX | | 0.02 | 1.3 | 4.2 | 1.5 | 37 | 23.3 | 21.6 | 3.2 | 1.6 | 3.6 |
| **USY-D500-Silica** | Hexane | | 0.03 | 0.8 | 2.9 | 1.5 | 47.4 | 26.1 | 17.1 | 3.0 | - | - |
|  | 10%DX | | 0.02 | 1.7 | 4.8 | 2.0 | 33.7 | 23.2 | 21.9 | 3.6 | 2.3 | 4.0 |

**Table S5:** Selectivity of the gaseous products each catalysts Cat and Cat D

|  |  | Gas (wt.%) | | | | | | | | | |
| --- | --- | --- | --- | --- | --- | --- | --- | --- | --- | --- | --- |
|  |  | H_2_ | CH_4_ | C_2_H_4_ | C_2_H_6_ | C_3_H_8_ | C_3_H_6_ | C_4_H_10_ | C_4_H_8_ | CO | CO_2_ |
| **Cat** | n-hexane | 0.1 | 3.4 | 7.2 | 3.3 | 53.2 | 17.7 | 13.1 | 2.0 | 0.0 | 0.0 |
|  | 30%DX | 0.1 | 6.4 | 8.2 | 4.8 | 35.0 | 20.2 | 11.2 | 2.8 | 5.0 | 6.0 |
| **Cat D** | n-hexane | 0.1 | 3 | 8 | 3 | 50.5 | 19.2 | 13 | 2.5 | 0.0 | 0.0 |
|  | 30%DX | 0.1 | 11.2 | 10.4 | 6.0 | 15.1 | 25.3 | 5.0 | 5.1 | 12.0 | 10.0 |

**Supplementary Texts**

**T****ext S1-** N-hexane quantitative analysis

The n-hexane amount was measured through calibration with an internal standard by a gas chromatograph coupled to a flame ionization detector (GCFID). Solutions containing 25, 50, 75 and 100 µL of n-hexane and 975, 950, 925 and 900 µL of acetone were prepared. Finally, 100 µL of n-hexadecane was added as an internal standard to each previously prepared standard solution. The sample solutions were prepared by adding 100 µL of sample to a 900 µL of acetone and 100 µL of n-hexadecane. Each compound (n-hexane, acetone and n-hexadecane) and samples were weighted to obtain the percentage weight of each substance on its respective solutions.

It was plotted a graphic whose ordinate axis was assigned as the ratio of areas of n-hexane and n-hexadecane multiplied by weight percentage of n-hexadecane (RA_C6_ x wt.%_C16_/RA_C16_) and the abscissa was assigned as the percentage weight of n-hexane (wt.%_C6_) (Supplementary Figure S5).

**Figure S5:** Calibration curve used to determine the n-hexane percentage weight on different samples.

The calibration curve (y = 1.0986x - 0.1915 ; R² = 0.9998) was generated by linear regression of points plotted on Supplementary Figure S5. Thus, the yield (wt.%_RP_) and mass (m_RP_) of reaction products on liquid phase were calculated as described in equations 8 and 9, respectively.

$wt.\% rp =100- \%wt.C6 (equation 8)$

$$m_{RP} =\frac{{wt.\%}_{RP}}{100} x M_{RP} (equation 9)$$

where,

M_RP_ = mass of liquid phase obtained at the end of reaction
